# Daily Low-Level Red Light for Spherical Equivalent Error and Axial Length in Children With Myopia: A Randomized Clinical Trial

**DOI:** 10.1001/jamaophthalmol.2024.0801

**Published:** 2024-04-25

**Authors:** Kai Cao, Lei Tian, Dong-Li Ma, Shi-Qiang Zhao, Ao Li, Zi-Bing Jin, Ying Jie

**Affiliations:** 1 Beijing Institute of Ophthalmology, Beijing Tongren Hospital, Capital Medical University, Beijing, China; 2 Beijing Tongren Eye Center, Beijing Tongren Hospital, Capital Medical University, Beijing, China

## Abstract

**Importance:**

Treatments are needed to slow progression of or reduce incidence of myopia.

**Objective:**

To evaluate the efficacy and safety of daily 650-nm low-level red light (LLRL) for myopia treatment.

**Design, Setting, and Participants:**

Single-masked, randomized clinical trial at 1 site in China. Baseline measurements were completed from August to September 2021. Participants were children aged 6 to 12 years with spherical equivalent error (SER) of −6 diopters (D) to 3 D. Data were analyzed from March to July 2023.

**Interventions:**

Irradiation daily with 650-nm LLRL for 3 minutes twice daily 4 or more hours apart or no intervention.

**Main Outcomes and Measures:**

Primary outcomes were changes in cycloplegia SER and axial length (AL) at 6- and 12-month follow-up visits. Safety was assessed on masked fundus photograph evaluations.

**Results:**

A total of 336 children were randomly allocated into the LLRL group or control group in a 1:1 ratio. The control group contained 86 female patients (51.2%), and the treatment group contained 90 female patients (53.6%). The mean (SD) age, SER, and AL were 9.0 (1.9) years, −1.3 (1.5) D, and 23.8 (1.0) mm for all patients. A total of 161 (95.8%) in the LLRL group and 159 (94.6%) in the control group returned for the 6-month follow-up. A total of 157 (93.5%) in the LLRL group and 152 (90.5%) in the control group returned for the 12-month follow-up. Mean (SD) changes in SER were 0.15 (0.16) D and −0.26 (0.21) D for the LLRL group and the control group, respectively (difference, −0.41 D; 95% CI, −0.48 to −0.34 D; *P* < .001), at 6 months and 0.24 (0.27) D and −0.65 (0.33) D for the LLRL group and the control group, respectively (difference, −0.89 D; 95% CI, −0.95 to −0.83 D; *P* < .001), at 12 months. Mean (SD) changes in AL were −0.06 (0.08) mm and 0.13 (0.12) mm for the LLRL group and control group, respectively (difference, 0.19 mm; 95% CI, 0.16 to 0.22 mm; *P* < .001), at 6 months and −0.11 (0.10) mm and 0.26 (0.16) mm for the LLRL group and control group, respectively (difference, 0.37 mm; 95% CI, 0.34 to 0.40 mm; *P* < .001). Masked fundus photograph review did not identify retinal changes in either group.

**Conclusions and relevance:**

These findings suggest daily use of 650-nm LLRL for 1 year can slow progression of SER and AL without safety concerns identified. Confirmation of these findings at independent sites seems warranted, as well as determining whether these effects can be sustained with or without continued treatment and whether LLRL has any effect on pathological myopia.

**Trial Registration:**

ChiCTR2200058963

## Key Points

**Question:**

What is the effect of daily 650-nm low-level red light (LLRL) on spherical equivalent error (SER) and on axial length (AL)?

**Findings:**

In this randomized clinical trial of 336 children at a single center where the device is patented, the mean change in SER was almost 1 diopter more myopic, with about one-third of a millimeter greater axial length in the control compared with the LLRL group, without adverse effects noted in the retina.

**Meaning:**

These findings suggest that daily use of 650-nm LLRL for 1 year can slow the progression of SER and AL without safety concerns identified; confirmation of these findings at independent sites seems warranted.

## Teaser

This randomized clinical trial evaluates the efficacy and safety of daily 650-nm low-level red light for myopia treatment among children aged 6 to 12 years in China.

## Introduction

An increasing amount of attention has been devoted to interventions for myopia control in children, among which low-level red light (LLRL)^1,2,3,4,5,6^ has recently attracted widespread interest. LLRL works by concentrating a specific band of light, typically 650 nm, into a laser beam to irradiate the retina.^1,2,3,4,5,6^

LLRL at 650 nm has been shown to help slow myopia progression without major safety concerns.^1,2,3,4,5,6^ However, it remains unclear whether LLRL has similar effects on children without myopia, for whom interventions are usually not provided due to ethical considerations. Nevertheless, some children with emmetropia or hyperopia may develop myopia rapidly,^7,8^ and their hyperopia reserves are depleted quickly compared with their peers.^9,10^ In addition, a previous study reported a dose-response relationship between treatment compliance with LLRL and efficacy in myopia control.^1^ It is worthwhile to explore whether using LLRL 7 days a week is more effective than using it 5 days a week, as adopted in previous studies.^1,5^

In the present study, we evaluated the effects of daily use of 650-nm LLRL over the course of 1 year. The participants included children with myopia, emmetropia, and low hyperopia.

## Methods

### Study Design

This was a single-masked, single-center, randomized clinical trial. Participants were recruited via online media or via the outpatient service. All recruited children were invited to the outpatient clinic of Beijing Tongren Hospital in Beijing, China, for qualification assessment. A total of 451 registered participants were assessed, and 336 children were determined to be eligible. The participants were randomly assigned to the LLRL group or control group at a 1:1 ratio. The recruitment period was from August 12 to September 3, 2021. The present study will continue until the 24-month follow-up is completed (see study protocol in Supplement 2).

This study was approved by the ethics committee at Beijing Tongren Hospital, Capital Medical University. Beijing Tongren Hospital has a patent for the LLRL device; no external advice or oversight of the ethics committee was provided from outside. Informed written consent was obtained from children’s parents, and the participants received free ophthalmic examinations and free device usage. This clinical trial adhered to the tenets of the Declaration of Helsinki. This study followed the Consolidated Standards of Reporting Trials (CONSORT) reporting guideline.

### Inclusion and Exclusion Criteria

Inclusion criteria were as follows: (1) aged 6 to 12 years; (2) cycloplegic spherical equivalent error (SER) of between −6 diopters (D) and 3 D in both eyes; for children without myopia, the change in SER was −0.75 D or less in the last year; (3) astigmatism of 2.5 D or less; and (4) patients who were willing to participate in the study and signed the informed consent form.

Exclusion criteria were as follows: (1) previously received other myopia interventions or stopped at 3 or fewer months, including atropine or orthokeratology lenses; (2) anisometropia of 1.5 D or greater, strabismus, or amblyopia; (3) refractive media opacification (keratopathy, lens opacity, and so forth); or (4) allergy to cycloplegia drugs.

### Intervention and Study Procedures

Children in the LLRL group were given a head-worn device^11^ with a 650-nm single-wavelength light source incorporated. This device was confirmed to be safe and was certified by the State Administration for Market Regulation of China. Children were expected to use the device 3 minutes twice daily 4 or more hours apart. In both groups, children with myopia could wear single-vision spectacle lenses. No other intervention was provided to the control group.

The device is automatically connected to the internet once powered on, so the time of use can be accurately recorded. The intervention compliance was calculated as actual device using time divided by targeted device using time. Outcome measurements (including primary and secondary outcomes and fundus interpretation) were completed by independent investigators (A.L. and K.C.) unaware of the grouping, and the statistician was also masked. Primary outcomes include changes in axial length (AL) and cycloplegia SER. SER was calculated from the dioptric powers of the sphere and half of the cylinder. Secondary outcomes include changes in choroid thickness (ChT), central corneal thickness, intraocular pressure, anterior chamber depth, flat keratometry (K1), steep keratometry (K2), and length thickness.

For safety evaluation, fundus photography and optical coherence tomography (OCT) were performed to assess the structure of the retina. Uncorrected distance visual acuity (UDVA) was measured to evaluate retinal function. Security assessment will pay attention to hemorrhage, exudation, retinal nerve fiber layer defect, and discontinuity of retina layer. The 2 primary outcomes and secondary outcomes were determined at 6- and 12-month follow-up visits.

The children’s pupils were dilated using tropicamide eye drops, and the refractive error was subsequently measured using an autorefractor (ARK-510A; Nidek Co Ltd). Myopia, moderate myopia, and high myopia were defined as SER between −3 D and −0.5 D, SER between −6 D and −3 D, and−6 D or less, respectively, in any eye. Ocular biological parameters were measured using an optical biometer (Lenstar LS 900; HAAG-STREIT AG).

The ChT was measured via the enhanced-depth imaging technique (Spectralis HRA+OCT; Heidelberg Engineering) at 9 locations in the fundus as follows: subfoveal, 1 mm, and 3 mm around the fovea (temporal, nasal, superior, and inferior). ChT referred to the distance between outer choroid sclera margin and retinal pigment epithelium-Bruch complex, and was measured automatically using the built-in software of OCT.^11^

Fundus photography was performed with a Canon retinal fundus camera (CR-DGI; Canon, Inc) after pupils were dilated. The interpretation of the fundus images was performed independently by 2 ophthalmologists from Beijing Tongren Hospital, and the final interpretation was given by another senior ophthalmologist (L.T.). The intraocular pressure was measured by a noncontact tonometer (Canon TX-20; Canon Inc).

### Statistical Analysis

Details of the sample size estimation and random allocation sequence generation were described in our previous article.^2^ *t* Tests with difference and 95% CIs were used for comparison on continuous variables between groups. Pearson correlation analysis was used for correlation analysis between changes in outcomes. Data analysis was performed for all randomly assigned children according to the intention-to-treat principle, before which a Markov chain Monte Carlo method was used to process the missing data (11 in the LLRL group and 16 in the control group were imputed). In addition, analyses were completed using data from children’s right eyes, except for the definitions of myopia (defined by person). All analysis was done using open-source R version 4.2.0 (R Project for Statistical Computing). The significance level was set to be 0.0125, 2-tailed, and due to interim analysis, it was adjusted to 0.011 after O’Briene Fleming α-spending adjustment. The *P* values were not adjusted for multiple analysis. Data were analyzed from March to July 2023.

## Results

### Participant Screening

**Figure 1.**
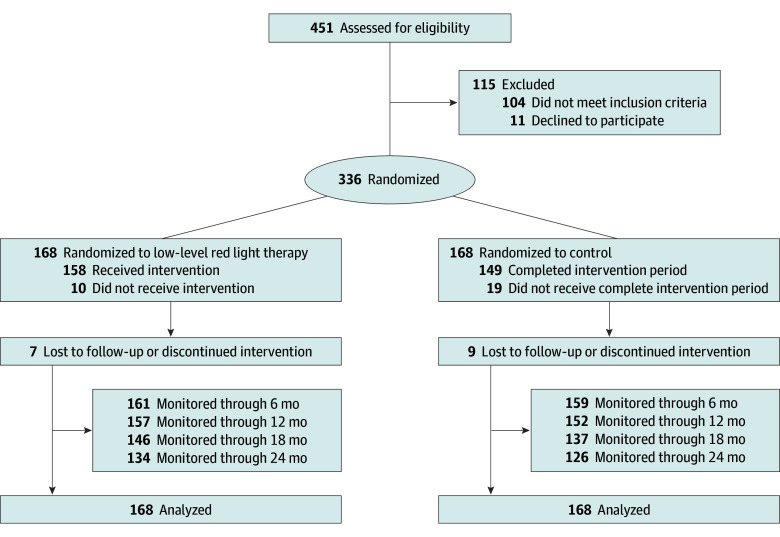
Participant Flow Diagram

Initially, 451 registered children were assessed for eligibility; 104 children did not meet the inclusion criteria, and 11 refused to sign the informed consent. The 336 participants included 224 with low to moderate myopia and 112 with emmetropia or low hyperopia. For 2 children, the SER was approximately 2 to 3 D; for the others, the SER ranged from −5.75 D to 1.375 D. The loss to follow-up rates were 4.2% (7 of 168) and 5.4% (9 of 168) for the LLRL group and control group at 6 months, and 6.5% (11 of 168) and 9.5% (16 of 168) for the LLRL group and control group at 12 months (Figure 1).

### Participants’ Baseline Characteristics

The mean (SD) age was 9.0 (1.9) years for all patients (9.0 [1.9] years for the control group and 9.1 [2.0] years for the treatment group). The control group contained 86 female patients (51.2%), and the treatment group contained 90 female patients (53.6%). The mean (SD) SER was −1.3 (1.5) D for all patients, including −1.3 (1.5) D for the control group and −1.4 (1.6) D for the treatment group. The mean AL was 23.8 (1.0) mm for all patients, including 23.8 (1.0) mm for the control group and 23.9 (1.1) mm for the treatment group. The details of the participants’ baseline information are shown in Table 1.

**Table 1.** Participants’ Baseline Characteristics

Characteristic	Participants, mean (SD)	
	Control (n = 168)	Treatment (n = 168)
Sex, No. (%)		
Female	86 (51.2)	90 (53.6)
Male	82 (48.8)	78 (46.4)
Age, y	9.0 (1.9)	9.1 (2.0)
Body mass index^a^	17.7 (3.7)	17.4 (3.8)
Spherical equivalent error, D		
Overall	−1.3 (1.5)	−1.4 (1.6)
Children with myopia	−2.1 (1.2)	−2.2 (1.2)
Children without myopia	0.3 (0.5)	0.2 (0.6)
Axial length, mm		
Overall	23.8 (1.0)	23.9 (1.1)
Children with myopia	24.2 (0.8)	24.3 (0.9)
Children without myopia	23.1 (0.7)	23.1 (0.8)
Intraocular pressure, mm Hg	15.4 (2.8)	14.9 (2.8)
Central corneal thickness, μm	546.3 (32.8)	543.9 (31.4)
Anterior chamber depth, mm	3.1 (0.3)	3.2 (0.3)
Length thickness, mm	3.4 (0.1)	3.4 (0.2)
Subfoveal choroid thickness, μm	299.3 (68.2)	291.4 (71.6)
K1, D	42.9 (1.3)	43.0 (1.4)
K2, D	44.1 (1.4)	44.2 (1.6)
Astigmatism, D	1.2 (0.5)	1.2 (0.6)

Abbreviations: D, diopter; K1, flat keratometry; K2, steep keratometry.

^a^ Body mass index is calculated as weight in kilograms divided by height in meters squared.

### Intervention Compliance

The device automatically connected to the internet once it was powered on, and thus, the duration of use could be accurately recorded. Without counting the 11 children who quit the trial or were lost to follow-up, the intervention compliance of children in the LLRL group ranged from 63% to 100% (median [IQR], 86% [72%-93%]).

### Changes in AL

The mean (SD) changes in AL were −0.06 (0.08) mm and 0.13 (0.12) mm for the LLRL group and control group, respectively (difference, 0.19 mm; 95% CI, 0.16 to 0.22 mm; *P* < .001), at 6 months and −0.11 (0.10) mm and 0.26 (0.16) mm for the LLRL group and control group, respectively, at 12 months (difference, 0.37 mm; 95% CI, 0.34 to 0.40 mm; *P* < .001) (Table 2).

**Table 2.** One-Year Change in Outcomes

Characteristic	Mean change (SD)		Mean difference (95% CI)	*P* value
	Control (n = 168)	Treatment (n = 168)		
All children (n = 336)				
AL, mm	0.26 (0.16)	−0.11 (0.10)	0.37 (0.34 to 0.40)	<.001
SER, D	−0.65 (0.33)	0.24 (0.27)	−0.89 (−0.95 to −0.83)	<.001
ChT, μm	−22.26 (12.05)	16.46 (18.15)	−38.72 (−42.02 to −35.41)	<.001
UDVA	−0.09 (0.32)	0.02 (0.36)	−0.11 (−0.18 to −0.04)	<.001
IOP, mm Hg	0.22 (1.53)	0.63 (1.88)	−0.41 (−1.21 to 0.39)	.31
CCT, μm	2.49 (4.61)	6.58 (5.54)	−4.09 (−14.75 to 6.57)	.45
AD, mm	0.08 (0.13)	0.06 (0.15)	0.01 (−0.02 to 0.05)	.38
LT, mm	−0.04 (0.12)	−0.04 (0.12)	−0.01 (−0.03 to 0.02)	.75
K1, D	−0.09 (0.75)	−0.16 (0.77)	0.07 (−0.09 to 0.23)	.38
K2, D	−0.04 (0.71)	−0.028 (0.75)	−0.01 (−0.17 to 0.14)	.86
Children with myopia (n = 224)				
AL, mm	0.27 (0.14)	−0.12 (0.11)	0.39 (0.35 to 0.43)	<.001
SER, D	−0.71 (0.30)	0.26 (0.29)	−0.97 (−1.05 to −0.89)	<.001
ChT, μm	−23.38 (9.24)	17.88 (19.42)	−41.26 (−45.26 to −37.26)	<.001
UDVA	−0.06 (0.29)	0.034 (0.37)	−0.09 (−0.18 to −0.01)	<.001
Children without myopia (n = 112)				
AL, mm	0.24 (0.18)	−0.09 (0.08)	0.33 (0.28 to 0.38)	<.001
SER, D	−0.51 (0.35)	0.20 (0.22)	−0.71 (−0.82 to −0.60)	<.001
ChT, μm	−20.02 (16.15)	13.63 (15.05)	−33.65 (−39.49 to −27.81)	<.001
UDVA	−0.16 (0.36)	−0.01 (0.33)	−0.15 (−0.28 to −0.02)	<.001

Abbreviations: AD, anterior chamber depth; AL, axial length; CCT, central corneal thickness; ChT, choroid thickness; D, diopter; IOP, intraocular pressure; K1, flat keratometry; K2, steep keratometry; LT, length thickness; SER, spherical equivalent error; UDVA, uncorrected distance visual acuity.

### Changes in Refractive Status

**Figure 2.**
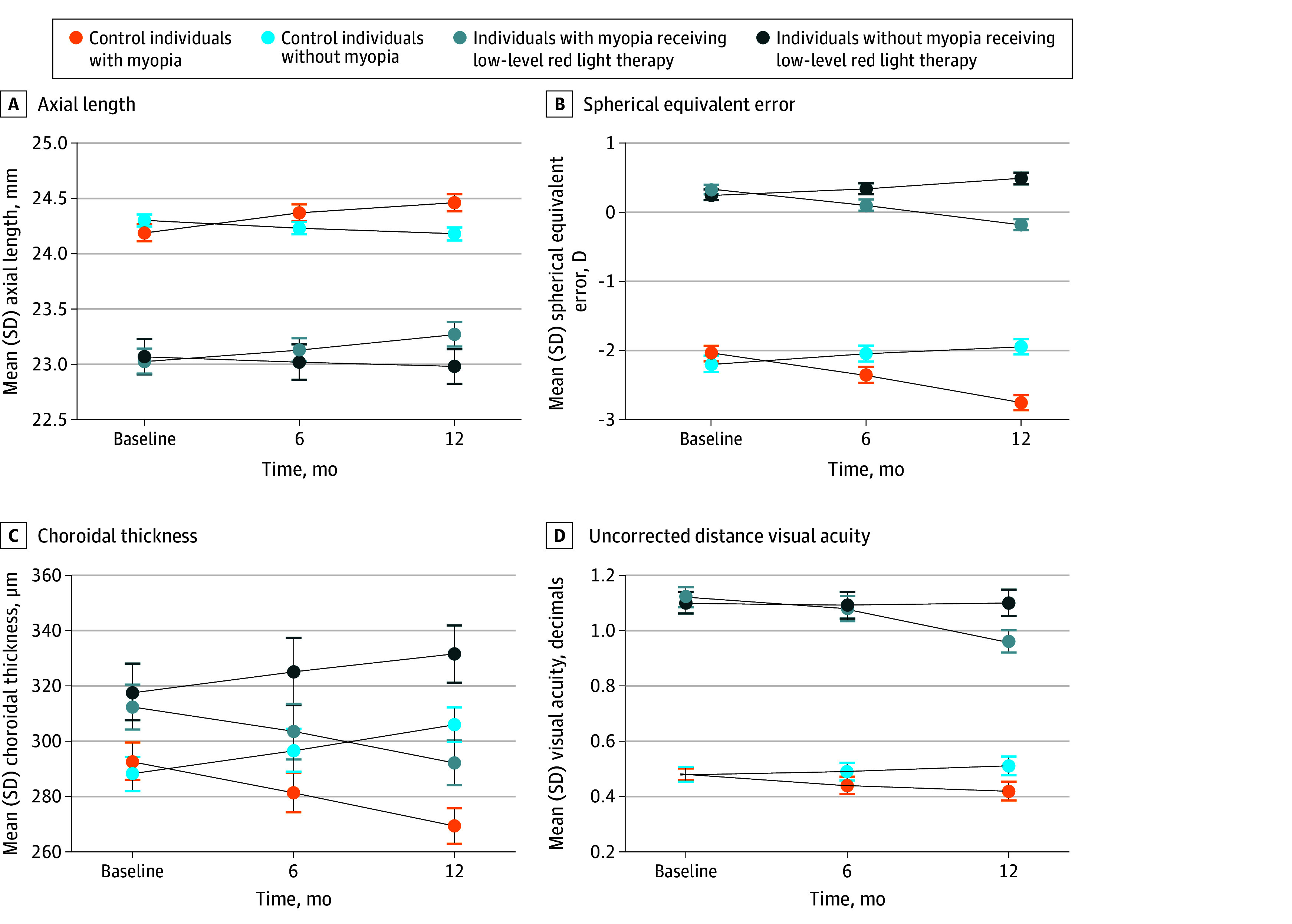
Line Plot of Outcomes Measured at Different Time Points

The mean (SD) changes in SER were 0.15 (0.16) D and −0.26 (0.21) D for the LLRL group and the control group, respectively (difference, −0.41 D; 95% CI, −0.48 to −0.34 D; *P* < .001), at 6 months and 0.24 (0.27) D and −0.65 (0.33) D for the LLRL group and the control group, respectively (difference, −0.89 D; 95% CI, −0.95 to −0.83 D; *P* < .001), at 12 months (for changing trend of AL and SER from baseline to 6- and 12-month follow-up, see Figure 2).

### Secondary Outcomes

The mean (SD) changes in subfoveal ChT in the control group and LLRL group were −22.26 (12.05) μm and 16.46 (18.15) μm, respectively (Table 2), and the mean difference was −38.72 μm (95% CI, −42.02 to −35.41 μm; *P* < .001). There were no differences in the following outcomes: intraocular pressure, central corneal thickness, anterior chamber depth, length thickness, K1, or K2. The results of eye fundus and OCT image analyses revealed no adverse event or damage to the retina.

### Other Outcomes

The mean (SD) changes in AL for the control and LLRL groups among children who had myopia at baseline were 0.27 (0.14) mm and −0.12 (0.11) mm, respectively (Table 2), and the mean difference was 0.39 mm (95% CI, 0.35 to 0.43 mm; *P* < .001). The mean (SD) changes in AL for the control and LLRL groups among children who did not have myopia at baseline were 0.24 (0.18) mm and −0.09 (0.08) mm, respectively (Table 2), and the mean difference was 0.33 mm (95% CI, 0.28 to 0.38 mm; *P* < .001).

The mean (SD) changes in the SER for the control and LLRL groups among children who had myopia at baseline were −0.71 (0.30) D and 0.26 (0.29) D, respectively (Table 2), and the mean difference was −0.97 D (95% CI, −1.05 to −0.89 D; *P* < .001). The mean (SD) changes in the SER for the control and LLRL groups among children who did not have myopia at baseline were −0.51 (0.35) D and 0.20 (0.22) D, respectively (Table 2), and the mean difference was −0.71 D (95% CI, −0.82 to −0.60 D; *P* < .001).

The mean (SD) changes in AL were −0.07 (0.08) mm and 0.55 (0.63) mm for the LLRL group and control group, respectively (difference, 0.62 mm; 95% CI, 0.51 to 0.73 mm; *P* < .001), at 24 months. The mean (SD) changes in SER were 0.12 (0.13) D and −1.42 (0.88) D for the LLRL group and control group, respectively (difference, −1.54 D; 95% CI, −1.68 to −1.40 D; *P* < .001), at 24 months.

The 1-year incidences of myopia in the LLRL group and the control group were 7.14% (4 of 56 patients) and 23.21% (13 of 56 patients), respectively. The incidence of myopia in the control group was greater than that in the LLRL group (16.07%; 95% CI, 2.66% to 29.35%; *P* = .02). The mean (SD) changes in UDVA in the control group and LLRL group were −0.09 (0.32) and 0.02 (0.36), respectively (Table 2), and the mean difference was −0.11 (95% CI, −0.18 to −0.04; *P* < .001).

eAppendix 1 in Supplement 1 describes subgroup analysis results for secondary outcomes. eFigure 1 in Supplement 1 presents correlation analysis of changes in primary and secondary outcomes in 4 subgroups. eFigure 2 in Supplement 1 fitted the regression model between the change in AL and the change in ChT. eTable 1 in Supplement 1 presents the results of per-protocol analysis. eTable 2, eTable 3, and eTable 4 in Supplement 1 present the results of subgroup analysis by gender, age, and refractive status at baseline, respectively. eFigure 3 in Supplement 1 presents the association between changes in primary outcomes and treatment compliance. eFigure 4 and eFigure 5 in Supplement 1 present changes in outcomes by time. eFigure 6 in Supplement 1 presents user compliance by month. eFigure 7 in Supplement 1 presents distribution of changes in primary outcomes at 2 follow-up time points. eAppendix 2 in Supplement 1 provides supplementary instructions on the use of equipment

## Discussion

The present study revealed that through 1 year of daily use of 650-nm LLRL, the mean changes in SER and AL were −0.11 mm and 0.24 D, respectively, and the mean change in subfoveal ChT was 16.46 μm. The interpretation of eye fundus and OCT image analyses revealed no adverse event or damage to the retina. The myopia incidence was 7.14%. The mean change in UDVA was 0.02.

There are dozens of effective methods for myopia control before LLRL, such as orthokeratology,^12,13^ atropine eye drops,^14,15^ peripheral defocus-modifying lenses,^16,17^ and outdoor time.^18,19^ Atropine was reported to be one of the most effective therapeutic options.^20^ However, the mean change in the SER of atropine-treated children was between −0.63 D per year and −0.16 per year.^21,22,23,24^ In contrast, the mean change in the SER in children receiving daily 650-nm LLRL intervention in the present study was 0.24 D per year. Another recent study reported similar results,^25^ where the mean changes in the SER were −0.03 D per year and −0.60 D per year for the LLRL group and 0.01% atropine group, respectively.^25^ Based on previously mentioned evidence, the effect of 650-nm LLRL may be stronger than that of other available interventions. However, the present trial did not directly compare various intervention measures, and further evidence is needed to support this conclusion.

Results of subgroup analysis on primary outcomes suggest interactions for the treatment group and baseline refractive status. The mean changes in AL and SER were 0.12 mm and 0.09 mm for children with myopia and were 0.26 D and 0.20 D for children without myopia. In terms of AL and SER, children with myopia benefit 30% or more from 650-nm intervention treatment than children without myopia.

There may be a dose-response effect between 650-nm LLRL use and myopia control. In a previous study^1^ that used 650-nm LLRL 5 days per week, the 1-year mean difference in AL between the treatment and control groups was 0.26 mm; however, in the present study, with a higher frequency of 7 days per week, the 1-year mean difference in AL was 0.37 mm. In all these studies, the participants were of similar age, the duration of a single intervention was the same (3 minutes), and the power of the laser entering the pupil was the same (0.29 mW). In a study by Jiang et al,^1^ the median compliance was 75%, and 14.2% of the patients had a compliance rate less than 50%. In the present study, the median compliance was 86%, and the lowest compliance was 63%. The higher compliance may explain why the effect in this study seems better than that in previous studies.^1,3^

LLRL has appeared to be safe when used for the treatment of retinal diseases and amblyopia decades ago.^26,27^ In this trial, 3 children reported seeing afterimages for a long time, and 4 children reported feeling strong light. Otherwise, no adverse event occurred. OCT and eye fundus images revealed no structural damage to the retina. Moreover, there was no decrease in the UDVA of children in the LLRL group, suggesting no functional damage. Considering that every intervention has its drawbacks (eg, atropine causes sensory discomfort, orthokeratology lenses cause corneal staining^28,29,30^ or microbial keratitis^31^), 650-nm LLRL may have a good safety profile if these results can be duplicated at sites independent of the institution that has a patent on the device used. However, long-term observation of the safety of this treatment is still necessary.

Previous studies revealed an association between AL and ChT^32,33,34,35^; the present study further demonstrated 34.5% of the shortened AL could be explained by thickened ChT. AL shortening not only occurs in LLRL treatment, but also in the treatment of atropine or orthokeratology lens.^36,37,38,39^ Anatomically, an increase in ChT mechanically pushes the retina forward, which naturally shortens the AL.^32,40^ Moreover, the choroid is a highly vascularized tissue, and changes in the ChBF may affect the AL.^41^ During myopia progression, the ChT usually becomes thinner, and the ChBF decreases,^42^ which further leads to ischemia and hypoxia of the choroid^41,43^; subsequently, the ChT becomes thinner, forming a vicious cycle. A reduction in ChBF causes an insufficient supply of oxygen and nutrients to the sclera, which upregulates hypoxia-inducible factor 1-α,^42,44^ leading to the transdifferentiation of a large number of fibroblasts into myofibroblasts in the sclera and the downregulation of fibroblast type I collagen,^45,46^ accelerating the progression of axial myopia. The use of 650-nanometer LLRL irradiation may help improve ChBF, thus reversing the vicious cycle mentioned above.

The 650-nm LLRL may be a preventive measure for myopia. Previously, few studies^47,48,49^ on myopia prevention were conducted because of ethical debates. Two recent studies^47,48^ explored the possibility of using atropine for myopia prevention and reported opposite findings. For 650-nm LLRL, a clinical trial reported a 54.1% reduction in incident myopia among children with premyopia within 12 months.^49^ In our study, the 1-year myopia incidences were 7.1% and 23.2% for the LLRL and control groups, respectively, which was a 69.4% reduction. For children without myopia but at high risk of myopia (eg, when the SER progresses more than −0.75 D per year), the 650-nm LLRL can be a potential treatment option; however, further ethical exploration is needed in this regard.

### Strengths and Limitations

The strengths of this study included its single-masked, randomized clinical trial design; the standardized measurement of refraction with cycloplegia; and the use of comprehensive outcome measurements, including AL, SER, ChT, and UDVA. More importantly, this trial included a representative sample of participants with myopia, emmetropia, or low hyperopia. Moreover, the use of a head-mounted device was tracked so that the compliance of the participants could be accurately assessed.

The limitations of this study include the lack of masking of participants. Additionally, this was a single-center trial, and it is unclear whether these results can be generalized to other areas in China, Asia, or the rest of the world. Furthermore, the follow-up duration was only 1 year. In addition, since this investigation was undertaken during the COVID-19 pandemic, it is unclear if these results may have been influenced by unique factors during that time that are relevant to the interpretation of these results, such as time spent outdoors during the pandemic or time spent on digital devices.

### Conclusions

Following 1 year of daily irradiation using 650-nm LLRL compared with no LLRL, the mean change in SER was almost 1 D more myopic, with about one-third of a millimeter greater axial length in the control compared with the LLRL group, without adverse effects noted in the retina. Confirmation of these findings at independent sites seems warranted, as well as determining whether these effects can be sustained with or without continued treatment and whether LLRL has any effect on pathological myopia.

## Notes

Supplement 1. **eAppendix 1.** Description of Subgroup Analysis Results for Secondary Outcomes **eFigure 1. **Correlation Analysis of Changes in Primary and Secondary Outcomes in 4 Subgroups **eFigure 2. **Scatter Plot of Changes in Axial Length and Changes in Choroid Thickness **eTable 2.** One-Year Change in Outcomes **eTable 3. **Subgroup Analysis of the 1-year Change in Outcomes by Gender **eTable 4. **Subgroup Analysis of the 1-Year Change in Outcomes by Age **eTable 5.** Subgroup Analysis of the 1-year Change in Outcomes by Different Refractive Status at Baseline **eFigure 3. **Matrix Scatter Plot of Changes in Primary Outcomes and Treatment Compliance **eFigure 4. **Line Plot of Changes in Outcomes From Baseline to 6-Month Follow-Up and From Baseline to 12-Month Follow-Up **eFigure 5. **Line Plot of Changes in Outcomes From Baseline to 6-Month Follow-Up and From 6-Month Follow-Up to 12-Month Follow-Up **eFigure 6. **Box-Plot of User Compliance by Month **eFigure 7. **Distribution of Changes in Primary Outcomes at 2 Follow-Up Time **eAppendix 2. **Supplementary Instructions on the Use of Equipment

Supplement 2.
Trial Protocol

Supplement 3.
Data Sharing Statement
