# Supplementary material for: Daily Low-Level Red Light for Spherical Equivalent Error and Axial Length in Children With Myopia: A Randomized Clinical Trial
Source: JAMA Ophthalmol. 2024 Apr 25;142(6):560–7. doi: 10.1001/jamaophthalmol.2024.0801 (PMC11046409; doi:10.1001/jamaophthalmol.2024.0801)
Supplement: Supplement 1. — eAppendix 1. Description of Subgroup Analysis Results for Secondary Outcomes eFigure 1. Correlation Analysis of Changes in Primary and Secondary Outcomes in 4 Subgroups eFigure 2. Scatter Plot of Changes in Axial Length and Changes in Choroid Thickness eTable 2. One-Year Change in Outcomes eTable 3. Subgroup Analysis of the 1-year Change in Outcomes by Gender eTable 4. Subgroup Analysis of the 1-Year Change in Outcomes by Age eTable 5. Subgroup Analysis of the 1-year Change in Outcomes by Different Refractive Status at Baseline eFigure 3. Matrix Scatter Plot of Changes in Primary Outcomes and Treatment Compliance eFigure 4. Line Plot of Changes in Outcomes From Baseline to 6-Month Follow-Up and From Baseline to 12-Month Follow-Up eFigure 5. Line Plot of Changes in Outcomes From Baseline to 6-Month Follow-Up and From 6-Month Follow-Up to 12-Month Follow-Up eFigure 6. Box-Plot of User Compliance by Month eFigure 7. Distribution of Changes in Primary Outcomes at 2 Follow-Up Time eAppendix 2. Supplementary Instructions on the Use of Equipment [file jamaophthalmol-e240801-s001.pdf]

Supplemental Online Content

Cao K, Tian L, Ma DL, et al. Daily low-level red light for spherical equivalent error and axial length in children with myopia. *JAMA Ophthalmol*. Published online April 18, 2024. doi:10.1001/jamaophthalmol.2024.0801

**eAppendix 1.** Description of Subgroup Analysis Results for Secondary Outcomes
**eFigure 1.** Correlation Analysis of Changes in Primary and Secondary Outcomes in 4 Subgroups
**eFigure 2.** Scatter Plot of Changes in Axial Length and Changes in Choroid Thickness
**eTable 1.** One-Year Change in Outcomes
**eTable 2.** Subgroup Analysis of the 1-year Change in Outcomes by Gender
**eTable 3.** Subgroup Analysis of the 1-Year Change in Outcomes by Age
**eTable 4.** Subgroup Analysis of the 1-year Change in Outcomes by Different Refractive Status at Baseline
**eFigure 3.** Matrix Scatter Plot of Changes in Primary Outcomes and Treatment Compliance
**eFigure 4.** Line Plot of Changes in Outcomes From Baseline to 6-Month Follow-Up and From Baseline to 12-Month Follow-Up
**eFigure 5.** Line Plot of Changes in Outcomes From Baseline to 6-Month Follow-Up and From 6-Month Follow-Up to 12-Month Follow-Up
**eFigure 6.** Box-Plot of User Compliance by Month
**eFigure 7.** Distribution of Changes in Primary Outcomes at 2 Follow-Up Time
**eAppendix 2.** Supplementary Instructions on the Use of Equipment

This supplemental material has been provided by the authors to give readers additional information about their work.

© 2024 Cao K et al. *JAMA Ophthalmology.*

eAppendix 1. Description of Subgroup Analysis Results for Secondary Outcomes

There are four subgroups by treatment and baseline refraction, myopic children in the LLRL group (TM), nonmyopic children in the LLRL group (TN), myopic children in the control group (CM), nonmyopic children in the control group (CN).

The mean change in subfoveal ChT for CM and TM subgroups were -23.38 µm and 17.88 µm (Table2), respectively, the mean difference was -41.26 (95%CI: -45.26 to -37.26) µm, p<.001. The mean change in subfoveal ChT for CN and TN subgroups were -20.02 µm and 13.63 µm (Table2), respectively, the mean difference was -33.65 (95%CI: -39.49 to -27.81) µm, p<.001.

The mean change in UDVA for CM and TM subgroups were -0.059 and 0.034 (Table2), respectively, the mean difference was -0.093 (95%CI: -0.181 to -0.005), p<.001. The mean change in UDVA for CN and TN subgroups were -0.16 and -0.01 (Table2), respectively, the mean difference was -0.15 (95%CI: -0.28 to -0.02) µm, p<.001.

© 2024 Cao K et al. *JAMA Ophthalmology.*

Correlation analysis of outcomes

There was a significant negative correlation between changes in AL and changes in SER (r=0.93, p<.001), a significant negative correlation between changes in AL and changes in subfoveal ChT (r=-0.81, p<.001), and a significant positive correlation between changes in SER and changes in subfoveal ChT (r=0.82, p<.001).

We further explored the association between changes in each outcome in four subgroups (see eFigure1).

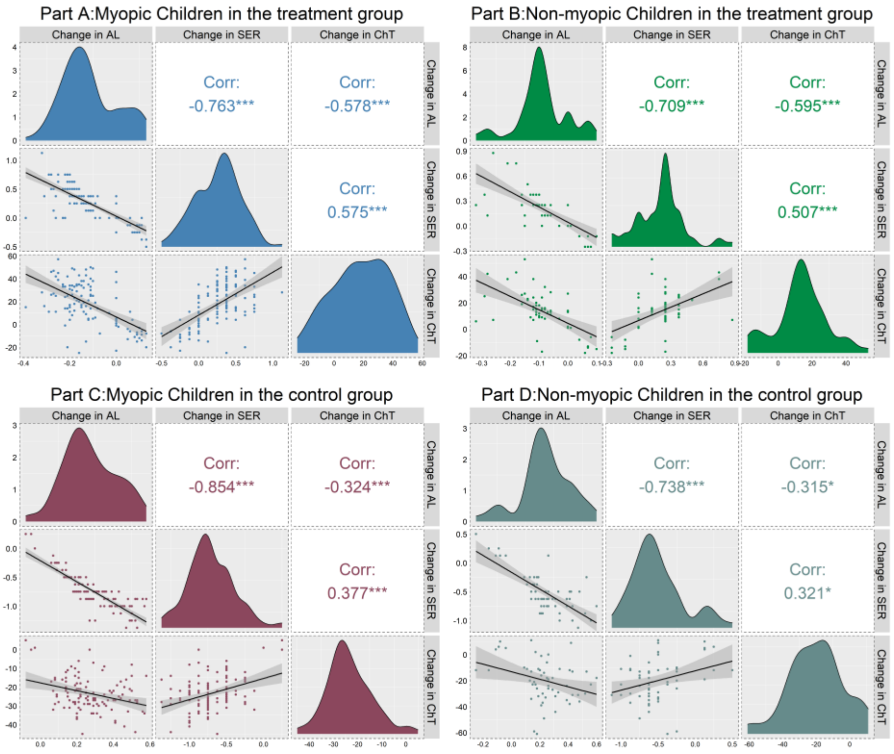


eFigure 1. Correlation Analysis of Changes in Primary and Secondary Outcomes in 4 Subgroups

For subgroup TM, TN, CM, and CN, the correlations coefficients of between changes in AL and changes in SER were -0.76 (p<.001), -0.71 (p<.001), -0.85 (p<.001), and -0.74 (p<.001), respectively.

The correlation coefficients of between changes in AL and changes in ChT were -0.58 (p<.001), -0.60 (p<.001), -0.32 (p<.001), and -0.32(p<.001), respectively for subgroup TM, TN, CM, and CN.

The correlation coefficients of between changes in SER and changes in ChT were 0.58 (p<.001), 0.51 (p<.001), 0.38 (p<.001), and 0.32 (p<.001), respectively for subgroup TM, TN, CM, and CN.

We fitted the regression model between the change in AL and the change in ChT, the results showed that the R square of the model was 34.5%, meaning that 34.5% of the AL shortening could be explained by the increase of ChT (eFigure2).

The association between changes in AL and changes in ChT could be described by the following formula:

Changes in AL= -0.0504 - 0.00352* changes in ChT (R-square of 34.5%)

© 2024 Cao K et al. *JAMA Ophthalmology.*

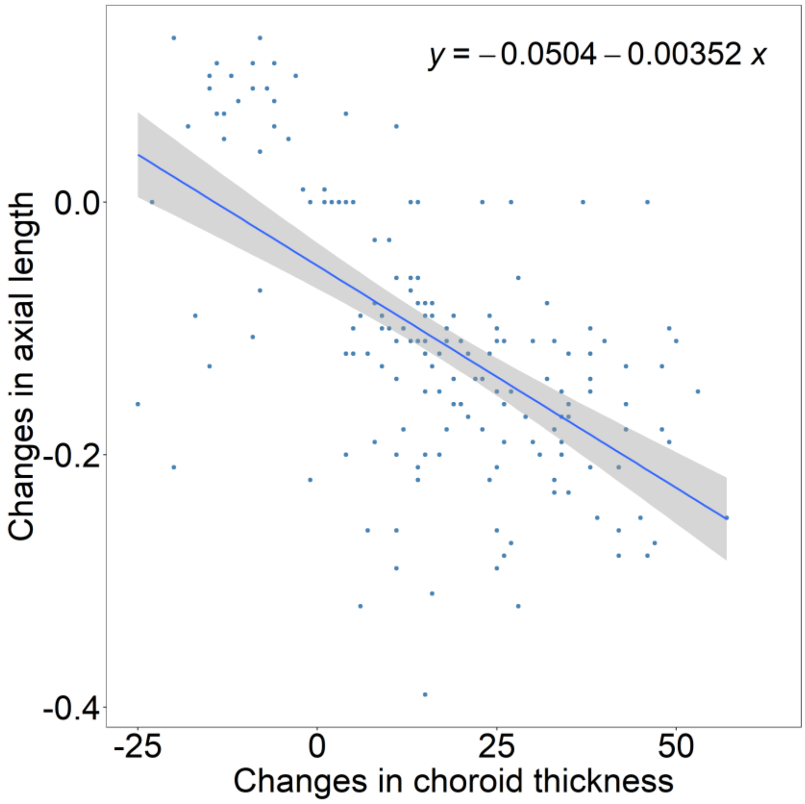


eFigure 2. Scatter Plot of Changes in Axial Length and Changes in Choroid Thickness

© 2024 Cao K et al. *JAMA Ophthalmology.*

Results of Per-protocol analysis

eTable 1. One-Year Change in Outcomes

| Characteristic | Control N = 152 | Treatment N = 157 | Mean difference (95%CI) | p-value |
|---|---|---|---|---|
| All children (N=309) | | | | |
| Change in AL (mm) | 0.26 ± 0.16 | -0.11 ± 0.11 | 0.37 (0.34 to 0.40 ) | <.001 |
| Change in SER (D) | -0.63 ± 0.34 | 0.25 ± 0.28 | -0.88 (-0.95 to -0.81 ) | <.001 |
| Change in ChT (μm) | -22.11 ± 12.31 | 16.87 ± 18.33 | -38.98 (-42.47 to -35.49 ) | <.001 |
| Change in UDVA | -0.10 ± 0.33 | 0.03 ± 0.36 | -0.13 (-0.21 to -0.05 ) | <.001 |
| Change in IOP (mmHg) | -0.05 ± 3.48 | 0.66 ± 3.84 | -0.71 (-1.53 to 0.11 ) | .09 |
| Change in CCT (μm) | 2.60 ± 4.53 | 7.28 ± 5.40 | -4.68 (-16.08 to 6.72 ) | .44 |
| Change in AD (mm) | 0.07 ± 0.13 | 0.06 ± 0.15 | 0.01 (-0.02 to 0.04 ) | .51 |
| Change in LT (mm) | -0.04 ± 0.12 | -0.04 ± 0.12 | 0.00 (-0.03 to 0.03 ) | .95 |
| Change in K1 (D) | -0.09 ± 0.78 | -0.14 ± 0.76 | 0.05 (-0.12 to 0.22 ) | .52 |
| Change in K2 (D) | -0.03 ± 0.72 | -0.02 ± 0.75 | -0.01 (-0.17 to 0.15 ) | .92 |
| Myopic children (N=207) | | | | |
| Change in AL (mm) | 0.27 ± 0.15 | -0.12 ± 0.11 | 0.39 (0.35 to 0.43 ) | <.001 |
| Change in SER (D) | -0.70 ± 0.32 | 0.27 ± 0.30 | -0.97 (-1.05 to -0.89 ) | <.001 |
| Change in ChT (μm) | -22.89 ± 9.32 | 17.96 ± 19.85 | -40.85 (-45.10 to -36.60 ) | <.001 |
| Change in UDVA | -0.07 ± 0.30 | 0.05 ± 0.38 | -0.12 (-0.21 to -0.03 ) | <.001 |
| Non-myopic children (N=102) | | | | |
| Change in AL (mm) | 0.23 ± 0.18 | -0.09 ± 0.09 | 0.32 (0.27 to 0.37) | <.001 |
| Change in SER (D) | -0.49 ± 0.35 | 0.21 ± 0.23 | -0.70 (-0.82 to -0.58) | <.001 |
| Change in ChT (μm) | -20.50 ± 16.86 | 14.67 ± 14.72 | -35.17 (-41.40 to -28.94) | <.001 |
| Change in UDVA | -0.17 ± 0.38 | -0.02 ± 0.33 | -0.15 (-0.29 to -0.01) | <.001 |

© 2024 Cao K et al. *JAMA Ophthalmology.*

eTable 2. Subgroup Analysis of the 1-year Change in Outcomes by Gender

| Characteristic | Control N = 168 | Treatment N = 168 | Mean difference (95%CI) | p-value |
|---|---|---|---|---|
| Boys (N=160) | | | | |
| Change in AL (mm) | 0.27 ± 0.17 | -0.12 ± 0.11 | 0.39 (0.35 to 0.43) | <.001 |
| Change in SER (D) | -0.65 ± 0.34 | 0.26 ± 0.27 | -0.91 (-1.01 to -0.81) | <.001 |
| Change in ChT (μm) | -20.50 ± 11.64 | 15.56 ± 17.39 | -36.06 (-40.71 to -31.41) | <.001 |
| Change in UDVA | -0.07 ± 0.31 | 0.03 ± 0.34 | -0.10 (-0.20 to -0.001) | .046 |
| Change in IOP (mmHg) | 0.16 ± 3.25 | 0.75 ± 3.90 | -0.59 (-1.71 to 0.53) | .30 |
| Change in CCT (μm) | 0.77 ± 2.16 | 5.60 ± 6.92 | -4.83 (-21.11 to 11.45) | .56 |
| Change in AD (mm) | 0.07 ± 0.14 | 0.04 ± 0.11 | 0.03 (-0.008 to 0.068) | .12 |
| Change in LT (mm) | -0.04 ± 0.12 | -0.02 ± 0.10 | -0.02 (-0.05 to 0.01) | .42 |
| Change in K1 (D) | 0.01 ± 0.73 | -0.13 ± 0.82 | 0.14 (-0.10 to 0.38) | .25 |
| Change in K2 (D) | 0.00 ± 0.71 | 0.00 ± 0.78 | 0.00 (-0.23 to 0.23) | .99 |
| Girls (N=176) | | | | |
| Change in AL (mm) | 0.25 ± 0.14 | -0.10 ± 0.10 | 0.35 (0.31 to 0.39) | <.001 |
| Change in SER (D) | -0.64 ± 0.33 | 0.23 ± 0.28 | -0.87 (-0.96 to -0.78) | <.001 |
| Change in ChT (μm) | -23.93 ± 12.25 | 17.23 ± 18.85 | -41.16 (-45.88 to -36.44) | <.001 |
| Change in UDVA | -0.11 ± 0.34 | 0.01 ± 0.38 | -0.12 (-0.22 to -0.02) | 0.02 |
| Change in IOP (mmHg) | 0.26 ± 3.80 | 0.52 ± 3.87 | -0.26 (-1.40 to 0.88) | 0.66 |
| Change in CCT (μm) | 4.14 ± 6.18 | 7.42 ± 3.26 | -3.28 (-18.07 to 11.51) | 0.66 |
| Change in AD (mm) | 0.08 ± 0.13 | 0.08 ± 0.17 | 0.00 (-0.046 to 0.046) | 0.94 |
| Change in LT (mm) | -0.052 ± 0.12 | -0.056 ± 0.13 | 0.004 (-0.032 to 0.040) | 0.83 |
| Change in K1 (D) | -0.18 ± 0.77 | -0.19 ± 0.72 | 0.01 (-0.21 to 0.23) | 0.98 |
| Change in K2 (D) | -0.08 ± 0.71 | -0.05 ± 0.72 | -0.03 (-0.24 to 0.18) | 0.78 |

© 2024 Cao K et al. *JAMA Ophthalmology.*

eTable 3. Subgroup Analysis of the 1-Year Change in Outcomes by Age

| Characteristic | Control N = 168 | Treatment N = 168 | Mean difference (95%CI) | p-value |
|---|---|---|---|---|
| Age≤9 (N=199) | | | | |
| Change in AL (mm) | 0.27 ± 0.16 | -0.10 ± 0.10 | 0.37 (0.33 to 0.41) | <.001 |
| Change in SER (D) | -0.63 ± 0.36 | 0.22 ± 0.26 | -0.85 (-0.94 to -0.76) | <.001 |
| Change in ChT (µm) | -22.96 ± 12.30 | 15.76 ± 17.01 | -38.72 (-42.84 to -34.60) | <.001 |
| Change in UDVA | -0.10 ± 0.31 | -0.01 ± 0.35 | -0.09 (-0.18 to -0.005) | 0.07 |
| Change in IOP (mmHg) | 0.80 ± 3.69 | 1.06 ± 4.16 | -0.26 (-1.35 to 0.83) | 0.65 |
| Change in CCT (µm) | 2.24 ± 5.64 | 7.58 ± 3.35 | -5.34 (-18.57 to 7.89) | 0.43 |
| Change in AD (mm) | 0.099 ± 0.15 | 0.096 ± 0.15 | 0.003 (-0.038 to 0.044) | 0.88 |
| Change in LT (mm) | -0.072 ± 0.12 | -0.064 ± 0.13 | -0.008 (-0.042 to 0.026) | 0.66 |
| Change in K1 (D) | -0.12 ± 0.79 | -0.14 ± 0.73 | 0.02 (-0.19 to 0.23) | 0.85 |
| Change in K2 (D) | -0.10 ± 0.71 | -0.02 ± 0.73 | -0.08 (-0.28 to 0.12) | 0.45 |
| Age>9 (N=137) | | | | |
| Change in AL (mm) | 0.25 ± 0.15 | -0.12 ± 0.12 | 0.37 (0.33 to 0.41) | <.001 |
| Change in SER (D) | -0.66 ± 0.31 | 0.28 ± 0.30 | -0.94 (-1.04 to -0.84) | <.001 |
| Change in ChT (µm) | -21.39 ± 11.74 | 17.65 ± 20.04 | -39.04 (-44.76 to -33.32) | <.001 |
| Change in UDVA | -0.086 ± 0.33 | 0.075 ± 0.36 | -0.161 (-0.279 to -0.043) | .008 |
| Change in IOP (mmHg) | -0.51 ± 3.20 | -0.11 ± 3.23 | -0.40 (-1.50 to 0.70) | .46 |
| Change in CCT (µm) | 2.81 ± 3.07 | 4.85 ± 7.49 | -2.04 (-22.27 to 18.19) | .84 |
| Change in AD (mm) | 0.046 ± 0.11 | 0.004 ± 0.12 | 0.042 (0.004 to 0.08) | .03 |
| Change in LT (mm) | -0.01 ± 0.11 | 0.00 ± 0.09 | -0.01 (-0.042 to 0.022) | .49 |
| Change in K1 (D) | -0.05 ± 0.71 | -0.20 ± 0.83 | 0.15 (-0.12 to 0.42) | .27 |
| Change in K2 (D) | 0.03 ± 0.71 | -0.04 ± 0.77 | 0.07 (-0.18 to 0.32) | .60 |

eTable 5. Subgroup Analysis of the 1-year Change in Outcomes by Different Refractive Status at

© 2024 Cao K et al. *JAMA Ophthalmology.*

| Baseline | | | | |
|---|---|---|---|---|
| Characteristic | Control | Treatment | Mean difference (95%CI) | p-value |
| Moderate myopia | N = 31 | N = 28 | | |
| Change in AL (mm) | 0.24 ± 0.11 | -0.14 ± 0.11 | 0.38 (0.32 to 0.44) | <.001 |
| Change in SER (D) | -0.66 ± 0.23 | 0.32 ± 0.29 | -0.98 (-1.12 to -0.84) | <.001 |
| Change in ChT (µm) | -24.00 ± 8.40 | 14.61 ± 19.06 | -38.61 (-45.89 to -31.33) | <.001 |
| Change in UDVA | 0.00 ± 0.19 | 0.08 ± 0.34 | -0.08 (-0.22 to 0.06) | .24 |
| Change in IOP (mmHg) | 0.38 ± 2.53 | -0.54 ± 3.33 | 0.92 (-0.62 to 2.46) | .24 |
| Change in CCT (µm) | 5.30 ± 2.24 | 15.83 ± 3.38 | -10.53 (-24.63 to 3.57 ) | .14 |
| Change in AD (mm) | 0.04 ± 0.09 | 0.00 ± 0.14 | 0.04 (-0.02 to 0.10) | .19 |
| Change in LT (mm) | -0.023 ± 0.08 | 0.013 ± 0.11 | -0.036 (-0.085 to 0.013) | .15 |
| Change in K1 (D) | -0.19 ± 0.69 | -0.17 ± 0.85 | -0.02 (-0.42 to 0.38) | .95 |
| Change in K2 (D) | 0.01 ± 0.56 | 0.03 ± 0.90 | -0.02 (-0.40 to 0.36) | .90 |
| Low myopia | N = 81 | N = 84 | | |
| Change in AL (mm) | 0.28 ± 0.15 | -0.12 ± 0.11 | 0.40 (0.37 to 0.43) | <.001 |
| Change in SER (D) | -0.72 ± 0.32 | 0.25 ± 0.28 | -0.97 (-1.06 to -0.88) | <.001 |
| Change in ChT (µm) | -23.06 ± 9.53 | 20.21 ± 18.98 | -43.27 (-48.05 to -38.49) | <.001 |
| Change in UDVA | -0.07 ± 0.31 | 0.01 ± 0.39 | -0.08 (-0.19 to 0.03) | .14 |
| Change in IOP (mmHg) | 0.29 ± 3.76 | 0.56 ± 3.44 | -0.27 (-1.38 to 0.84) | .63 |
| Change in CCT (µm) | -1.07 ± 6.21 | 0.88 ± 7.30 | -1.95 (-22.35 to 18.45) | .85 |
| Change in AD (mm) | 0.038 ± 0.11 | 0.031 ± 0.11 | 0.007 (-0.028 to 0.042) | .69 |
| Change in LT (mm) | -0.011 ± 0.12 | -0.016 ± 0.09 | 0.005 (-0.027 to 0.037) | .73 |
| Change in K1 (D) | -0.164 ± 0.78 | -0.205 ± 0.84 | 0.041 (-0.210 to 0.292) | .74 |
| Change in K2 (D) | -0.11 ± 0.78 | -0.03 ± 0.69 | -0.08 (-0.31 to 0.15) | .48 |
| Emmetropia | N = 37 | N = 36 | | |
| Change in AL (mm) | 0.25 ± 0.17 | -0.07 ± 0.12 | 0.32 (0.25 to 0.39) | <.001 |
| Change in SER (D) | -0.55 ± 0.31 | 0.17 ± 0.30 | -0.72 (-0.87 to -0.57) | <.001 |
| Change in ChT (µm) | -23.22 ± 16.65 | 9.75 ± 16.22 | -32.97 (-40.95 to -24.99) | <.001 |
| Change in UDVA | -0.21 ± 0.37 | -0.07 ± 0.33 | -0.14 (-0.31 to 0.03) | .10 |
| Change in IOP (mmHg) | 0.09 ± 4.01 | 1.16 ± 5.03 | -1.07 (-3.27 to 1.13) | .34 |
| Change in CCT (µm) | 9.66 ± 3.21 | 10.11 ± 3.02 | -0.45 (-15.52 to 14.62) | .95 |
| Change in AD (mm) | 0.17 ± 0.17 | 0.13 ± 0.17 | 0.04 (-0.04 to 0.12) | .34 |
| Change in LT (mm) | -0.11 ± 0.13 | -0.10 ± 0.15 | -0.01 (-0.075 to 0.055) | .79 |
| Change in K1 (D) | -0.015 ± 0.54 | -0.083 ± 0.71 | 0.068 (-0.236 to 0.370) | .66 |
| Change in K2 (D) | 0.01 ± 0.61 | -0.03 ± 0.78 | 0.04 (-0.30 to 0.38) | .82 |
| Hyperopia | N = 19 | N = 20 | | |
| Change in AL (mm) | 0.24 ± 0.19 | -0.09 ± 0.06 | 0.33 (0.24 to 0.42) | <.001 |

© 2024 Cao K et al. *JAMA Ophthalmology.*

| Characteristic | Control | Treatment | Mean difference (95%CI) | p-value |
|---|---|---|---|---|
| Change in SER (D) | -0.47 ± 0.40 | 0.20 ± 0.15 | -0.67 (-0.85 to -0.49) | <.001 |
| Change in ChT (μm) | -16.33 ± 14.89 | 17.71 ± 13.40 | -34.04 (-42.55 to -25.53) | <.001 |
| Change in UDVA | -0.102 ± 0.36 | 0.104 ± 0.29 | -0.206 (-0.401 to -0.011) | .04 |
| Change in IOP (mmHg) | -0.04 ± 2.93 | 1.99 ± 3.61 | -2.03 (-4.03 to -0.03) | .047 |
| Change in CCT (μm) | 3.46 ± 1.23 | 5.00 ± 2.59 | -1.54 (-14.24 to 11.16) | .81 |
| Change in AD (mm) | 0.13 ± 0.12 | 0.17 ± 0.13 | -0.04 (-0.12 to 0.04) | .30 |
| Change in LT (mm) | -0.10 ± 0.09 | -0.11 ± 0.10 | 0.01 (-0.05 to 0.07) | .77 |
| Change in K1 (D) | 0.19 ± 0.89 | -0.11 ± 0.39 | 0.30 (-0.11 to 0.71) | .14 |
| Change in K2 (D) | 0.08 ± 0.70 | -0.13 ± 0.57 | 0.21 (-0.17 to 0.59) | .27 |

Association between changes in primary outcomes and treatment compliance

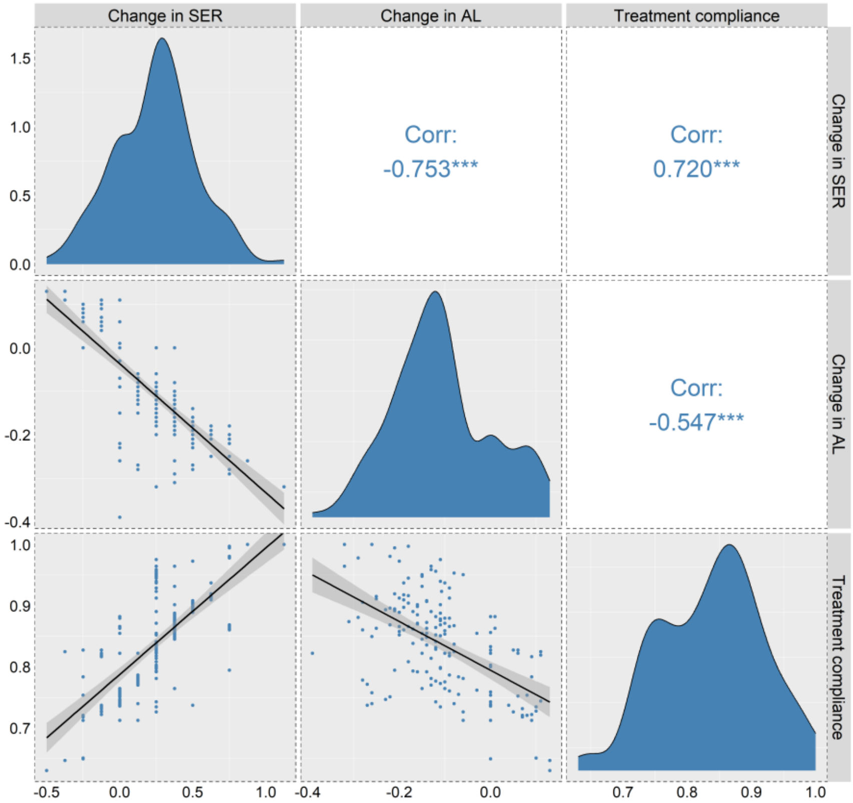


eFigure3 . Matrix Scatter Plot of Changes in Primary Outcomes and Treatment Compliance

© 2024 Cao K et al. *JAMA Ophthalmology.*

Line plot of changes in outcomes measured at different time points

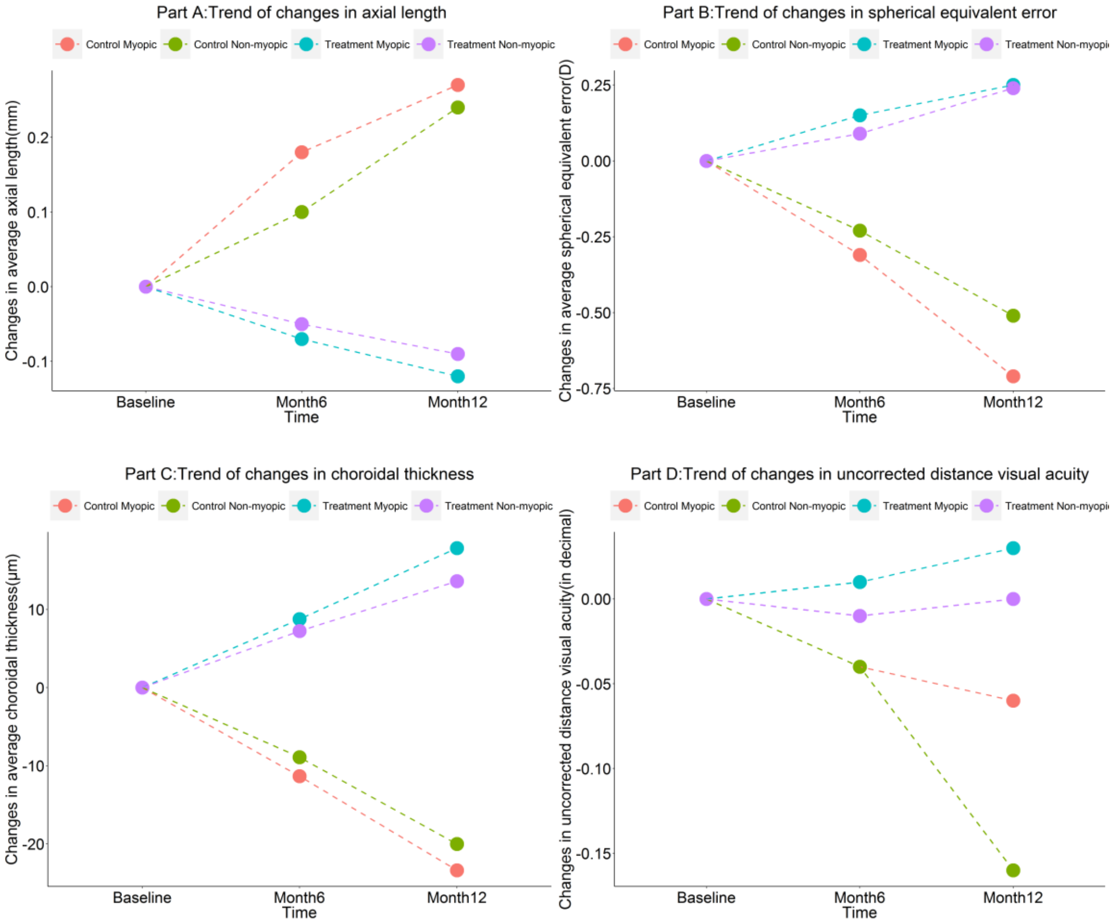


eFigure 4. Line Plot of Changes in Outcomes From Baseline to 6-Month Follow-Up and From Baseline to 12-Month Follow-Up

© 2024 Cao K et al. *JAMA Ophthalmology.*

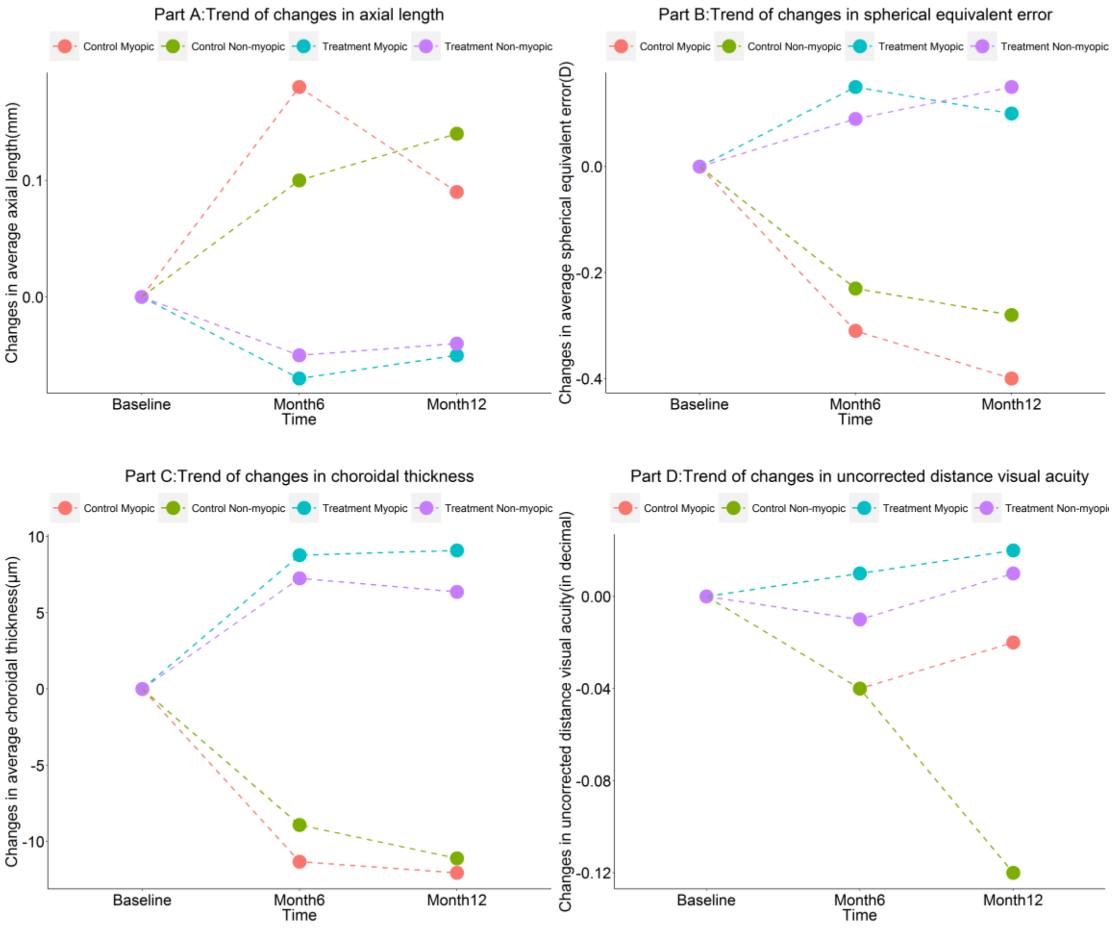


eFigure 5. Line Plot of Changes in Outcomes From Baseline to 6-Month Follow-Up and From 6-Month Follow-Up to 12-Month Follow-Up

Box-plot of user compliance by month

© 2024 Cao K et al. *JAMA Ophthalmology.*

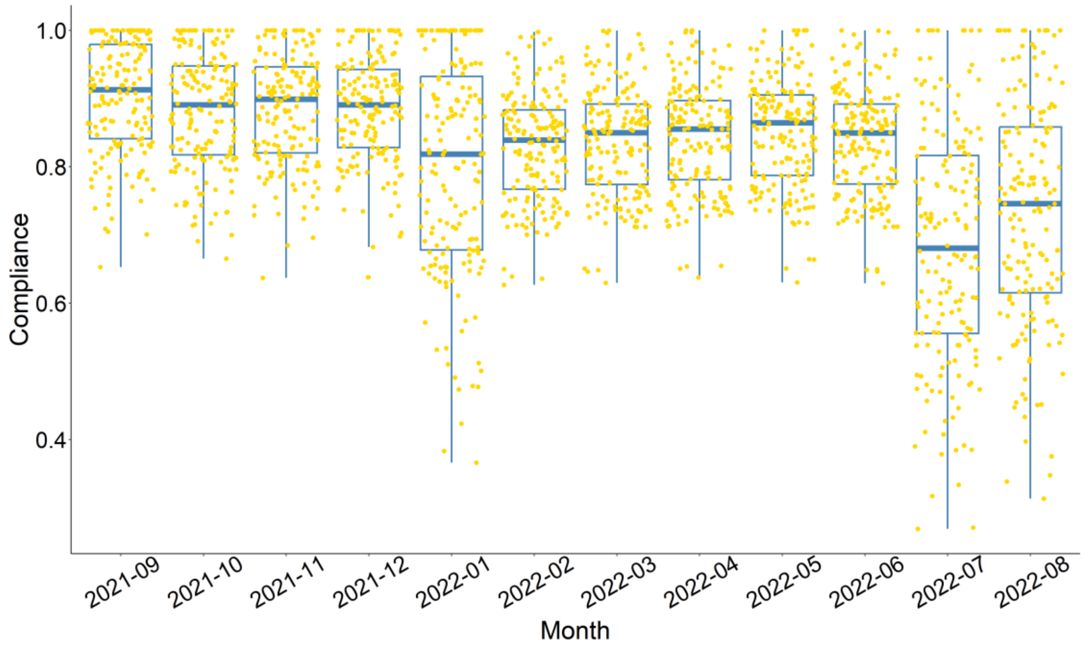


eFigure 6. Box-Plot of User Compliance by Month

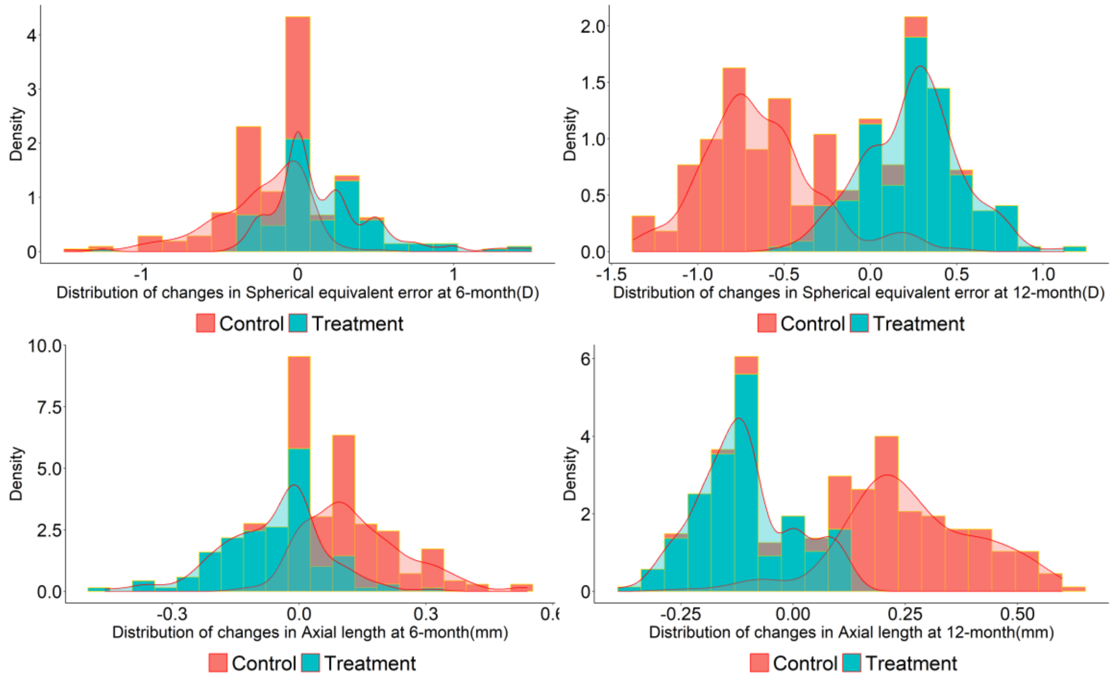


eFigure 7 Distribution of Changes in Primary Outcomes at 2 Follow-Up Time Points

Figure legends

© 2024 Cao K et al. *JAMA Ophthalmology.*

eFigure 1 Correlation analysis of changes in primary and secondary outcomes in four subgroups
eFigure2 Scatter plot of changes in axial length and changes in choroid thickness
eFigure3 matrix scatter plot of changes in primary outcomes and treatment compliance
eFigure 4 Line plot of changes in outcomes from baseline to 6-month follow-up and from baseline to 12-month follow-up
eFigure 5 Line plot of changes in outcomes from baseline to 6-month follow-up and from 6-month follow-up to 12-month follow-up
eFigure 6 Box-plot of user compliance by month
eFigure 7 Distribution of changes in primary outcomes at two follow-up time Points

eAppendix 2. Supplementary Instructions on the Use of Equipment

There was no recording to confirm that the study participant is the one who used the device instead of someone else.

However, we strive to ensure that each device is only used by one child from two aspects, firstly, we made it very clear at recruitment that each device can only be used by one child. Secondly, we asked the parents to supervise the device use.

Besides, the machine locks automatically after using it twice a day, so if two or more children are sharing the device, the treatment effect will inevitably be reduced, and will be reflected on axial length and spherical equivalent error.

© 2024 Cao K et al. *JAMA Ophthalmology.*
