# Supplementary material for: Daily Low-Level Red Light for Spherical Equivalent Error and Axial Length in Children With Myopia: A Randomized Clinical Trial
Source: JAMA Ophthalmol. 2024 Apr 25;142(6):560–7. doi: 10.1001/jamaophthalmol.2024.0801 (PMC11046409; doi:10.1001/jamaophthalmol.2024.0801)
Supplement: Supplement 2. — Trial Protocol [file jamaophthalmol-e240801-s002.pdf]

PROJECT TITLE

650nm Low-level Red-light for Myopia Control and Prevention in Children: A Randomized Controlled Trial

Principal investigator : Professor Ying Jie

Affiliation: Beijing Institute of Ophthalmology

Beijing Tongren Hospital, Capital Medical University

Date: 2021.06.30

Contents

Study protocol ... 3
1. Members of the Research Team ... 3
2. Study site ... 4
3. Background ... 4
4. Hypothesis ... 5
5. Study plan ... 5
    Study sites ... 6
    Study design ... 6
    Participants enrollment ... 6
    Inclusion and Exclusion Criteria ... 6
    Study Procedures ... 6
    Sample Size Estimation ... 7
    Randomization and Masking ... 8
    Visit plan and time frame ... 9
    Outcomes ... 9
    Outcomes Measurements ... 9
    Benefits of this study ... 10
    References: ... 10
6. Data management and statistical analysis ... 11
    Data collection and data cleaning ... 11
    Missing data handling ... 12
    Statistical analysis ... 12
        Data-set that used for analysis ... 12
        Statistical description and hypothesis testing ... 12
        Subgroup analyses ... 12
        Statistical Software ... 13
7. Ethical consideration ... 13
    Institutional Review Board Review and Informed Consent ... 13
    Subject Confidentiality ... 13

Study protocol

1. Members of the Research Team

Principal Investigator:

Name: Ying Jie, MD, PhD Professor, Clinician Scientist

Affiliation: Beijing Institute of Ophthalmology, Beijing Tongren Hospital, Capital Medical University

Email:jie_yingcn@aliyun.com

Address: No.17, Hougou alley, Dongcheng District, Beijing.

Co-Investigator 1:

Name: Lei Tian, MD, PhD Associate professor

Affiliation: Beijing Institute of Ophthalmology, Beijing Tongren Hospital, Capital Medical University

Address: No.17, Hougou alley, Dongcheng District, Beijing.

Co-Investigator 2:

Name: Kai Cao, MM Statistician

Affiliation: Beijing Institute of Ophthalmology, Beijing Tongren Hospital, Capital Medical University

Address: No.17, Hougou alley, Dongcheng District, Beijing.

Co-Investigator 3:

Name: Dong-Li Ma, MM Professor

Affiliation: Beijing Tongren Eye Center, Beijing Tongren Hospital, Capital Medical University

Address: No 1, DongJiaoMinXiang, DongCheng District, Beijing.

Study nurse:

Name: Li-Xin Lu, BM Head nurse

Affiliation: Beijing Tongren Eye Center, Beijing Tongren Hospital, Capital Medical University

Address: No 1, DongJiaoMinXiang, DongCheng District, Beijing.

Independent inspector:

Name: Changxi-Chen, BM Associate chief technician

Affiliation: Beijing Institute of Ophthalmology, Beijing Tongren Hospital, Capital Medical University

Address: No.17, Hougou alley, Dongcheng District, Beijing.

Independent inspector:

Name: Shi-Qiang Zhao, MM Associate chief technician

Affiliation: Beijing Tongren Eye Center, Beijing Tongren Hospital, Capital Medical University

Address: No 1, DongJiaoMinXiang, DongCheng District, Beijing.

Data Management:

Name: Ao Li

Affiliation: Beijing Tongren Eye Center, Beijing Tongren Hospital, Capital Medical University

Address: No 1, DongJiaoMinXiang, DongCheng District, Beijing.

2. Study site

Beijing Tongren Hospital, Capital Medical University

Beijing Institute of Ophthalmology

Beijing Tongren Hospital is a famous hospital in China, which specializes in ophthalmology and otolaryngology. Beijing Tongren Hospital ranks the top 5 hospital in ophthalmology for the latest ten years all over China.

Beijing Tongren Hospital is affiliated to Capital Medical University, consists of 10 Schools, 14 affiliated hospitals and 1 teaching institution. The university has over 9,000 enrolled students. It is the university with the strongest clinical medical level in Beijing, China.

Beijing Institute of Ophthalmology is a special research institute in ophthalmology. For the past 60 years, it has made great contributions to the treatment of ophthalmic diseases around the world, such as the treatment of trachoma (Chlamydia trachomatis was isolated from egg yolk sac of chicken embryo by Prof. Zhang Xiaolou and Prof. Tang Feifan), which has been awarded by the World Health Organization.

3. Background

Myopia is a worldwide public health issue. China alone has a myopic population that is over 0.4 billion

[1]. The prevalence of myopia is as high as 83.2% in Chinese university students who are aged between 16 to 26 years[2]. The myopia prevalence and incidence in China remain high, despite dozens of interventions that were reported to be effective for controlling this condition. Some of such interventions are orthokeratology, spectacles, intraocular lenses, refractive surgeries, atropine eye drops, as well as outdoor activities. The bottleneck lies in that each intervention has major drawbacks so none is suitable for myopia control and prevention at the population level.

Orthokeratology and spectacles are expensive, considering that the annual cost was reported to be around $198.30-$378.10 and $342.50[3], respectively. On the other hand, the annual cost of refractive surgeries was only $19.10. Intraocular lenses and refractive surgeries are invasive treatments, while intraocular lenses lead to endothelial cell density loss[4], posterior capsule opacification[5], and even retinal detachment[6]. For refractive surgeries, there is a risk of dry eye[7], keratitis[8, 9], ectasia[10], and even keratoconus[11], although the risk is relatively low[12, 13]. The main problem with atropine eye drops is that low dose has limited effects while high dose comes with obvious rebound effects once the treatment is stopped[14]. As for outdoor activity, the conflict between time cost and academic pressure is contradictory. Simply put, there is no perfect solution for the time being.

Recently, a new noninvasive solution has emerged, which is the 650nm low-level red-light (650 nm LLRL). The efficacy and safety of this method in slowing down myopia progression were preliminarily demonstrated in a randomized controlled trial[15]. The study also reported a significant reduction in axial length (AL) in 21.6% of children, suggesting that the 650nm LLRL may deduce the risk of myopia. However, the previous trial only included nearsighted children and did not fully demonstrated whether and by what extent 650nm LLRL reduces the risk of myopia.

In this case, we plan to design a large sample randomized controlled trial involving myopic and non-myopic subjects to update the findings, to give more representative findings.

4. Hypothesis

1) Hypothesis 1: Daily use of the 650nm low-level red-light (650 nm LLRL) slows down the myopia progression in Children.

2) Hypothesis 2: Daily use of the 650 nm LLRL reduces the risk of myopia onset in children.

3) Hypothesis 3: The daily use of 650 nm LLRL raises no damage to the fundus.

5. Study plan

Study sites

Beijing Tongren Hospital, affiliated to Capital Medical University in Beijing, China

Study design

A single-center, single-masked, randomized controlled trial.

Participants enrollment

Subjects will be enrolled through two ways, one way is by social media, we will post recruitment advertisements through WeChat APP (A native instant messaging tool in China), parents who are interested are expected to register by leaving their mobile phone number in the background of the APP. Another way is by putting up posters in ophthalmic clinics and conducting on-site enrollment. All registered children will be invited to the outpatient clinic of Beijing Tongren Hospital in Beijing, China for detailed ophthalmic examination to determine whether they are suitable for this trial.

The enrollment and screening duration depends on the sample size requirement and the inclusion and exclusion criteria.

Inclusion and Exclusion Criteria

Inclusion criteria: 1) Children aged 6 to 12 years. 2) Low to moderate myopia, emmetropia or low hyperopia. Low to moderate myopia: the cycloplegic spherical equivalent error (SER) of between -6D to -0.5D (>-6D,≤-0.5D) in both eyes. 3) Emmetropia or low hyperopia: the cycloplegic SER of between -0.5D to +3D (> -0.5D,≤+3D) in both eyes. For these participants, the decrease in SER in the past year should be 0.75D or more (change in SER ≤-0.75D). 4) Astigmatism of 2.5 D or less (≤2.5D). 5) Willing to participate in the study and sign the informed consent form.

Exclusion criteria: 1) Using other myopia interventions, or stopped using them for no more than three months, including but not limited to atropine eye drops or orthokeratology lens. 2) anisometropia (difference in sphere between two eyes was 1.5D or greater), strabismus, or amblyopia. 3) With refractive media opacification (keratopathy, lens opacity, etc.) 4) Allergy to cycloplegia drugs.

Study Procedures

Before enrollment and before any study-related procedures are performed, voluntary, written study-specific informed consent will be obtained from the participants' parents. Each signature on the informed consent form must be personally dated by the signatory. The investigator Ying Jie or his

assistants will also sign the informed consent form. A copy of the signed and dated informed consent form must be given to the participants. The source data must reflect that the informed consent is obtained before participation in the study.

Each child in the treatment group will be distributed a head-worn 650 nm low-level red-light device to take home. Their parents will be told how to use the equipment, and will be given instructions in papers and videos. To ensure the effectiveness, children will be encouraged to ensure that the utilization rate of the device is ≥90%. The device is connected to the network, and the time of use can be accurately recorded, so we can monitor the compliance of the subjects. Children in the treatment group are expected to use the device to irradiate the retina for six minutes every day, divided into two times of three minutes, with an interval of ≥4 hours.

Children in both groups are allowed to wear a single-vision spectacle lenses (SVS) if needed, without other intervention.

The light source used of the 650 nm low-level red-light is a single-wavelength weak laser red-light, with 650nm low intensity, and the radiation category is Class 1 light. Such light can be used safely in the eyes and this has been verified by the State Administration for Market Regulation. The light source is integrated on a head-mounted device (Product Name: Myopia and Amblyopia Treatment Apparatus. Product Model: YF020A. The Medical Device Registration No: 20212162067. Manufacturer: Hunan EnVan Technology Co.,Ltd.), which is a patented technology of Beijing Tongren Hospital (an optical device for the treatment of myopia and amblyopia, patent number is ZL202022533301.4). In both groups, children would be allowed to wear single-vision spectacles (SVS) if they have myopia. No additional intervention would be given to children the in the control group.

Sample Size Estimation

Due to that data on the myopia incidence of children receiving 650nm LLRL intervention is lacking, the following sample size estimation method will be used.

$$N=\frac{(Z_{1-\alpha/2}+Z_{1-\beta})^2\ (\sigma_1^2+\sigma_2^2)}{(\delta)^2}$$

N stands for sample size of each group.

$Z_{1-\alpha/2}$ and $Z_{1-\beta}$ dependent on Type I error and Type II error.

σ_1 and σ_2 represents the standardized deviation (SD) of primary outcomes of each group.

δ represents the difference in mean values of primary outcomes between the LLRL and control groups.

We consider the significance level of 0.025, and the power of no less than 0.9. Thus, $Z_{1-\alpha/2}$was 1.96, $Z_{1-\beta}$ was 1.28. According to the study by Jiang Y et al[15], the one-year mean changes in the AL of the LLRL and control groups were 0.13 (95% CI: 0.09, 0.17)mm and 0.38 (95% CI: 0.34, 0.42)mm, respectively. The one-year mean changes in the SER of the LLRL group and control group were -0.20 (95% CI: -0.29, -0.11)D and -0.79 (95% CI: -0.88, -0.69)D, respectively. According to the study by Xiong F et al[16], the six-month changes in the SER of the LLRL and control groups were 0.21±0.34D and -0.50±0.24D, respectively, while the mean changes in the AL were -0.06±0.15mm and 0.23±0.06mm, respectively.

Sample size estimation was done according to the changes in the AL or SER as reported by the above mentioned two studies, respectively. The estimated largest sample size was 39 for each group. The anticipated change in SER and AL for the control group were -0.75D and 0.35mm, and were -0.25D and 0.15mm for the RRLR group. The standard deviation was anticipated of no more than 0.55D for SER in each group and no more than 0.25mm for AL for each group (which was large enough, to make sure enough sample size). The power was set to be 0.9, the alpha was chosen as 0.05.

The sample size was calculated using PASS software, and the result was shown below.

Two-Sample T-Tests Assuming Equal Variance

Numeric Results for Two-Sample T-Test Assuming Equal Variance
Alternative Hypothesis: H1: δ = μ1 - μ2 ≠ 0

Target Power	Actual Power	N1	N2	N	μ1	μ2	δ	σ	Alpha
0.90	0.90515	39	39	78	-0.3	-0.7	0.5	0.6	0.025

Then considering a follow-up rate of 80%, the sample size was added to 49 for each group. Taking block randomization (length of block=8) into consideration, we added the sample size to 56 for each group. Last but not the least, considering that high myopia (related to the Hypothesis 3) is a relatively low-frequency event, we doubled the number of myopic children to 112 in each group, so at last the sample size of each group was set to be 168.

Randomization and Masking

The random sequence will be generated using the open-source R program (https://www.r-project.org/, version 4.2.0). The participants will share an equal probability to be assigned to either the treatment group or the control group, in a ratio of 1:1. We will use a stratified block randomization method, with

the block length of eight. Due to the distinct difference of interventions, both participants and researchers are aware of the allocation. In order to minimize the measurement bias, the measurement of outcomes will be completed by independent investigators. The statistician having access to the data will also be masked to the groupings.

Visit plan and time frame

Participants will be followed in every six months, there will be four visits in total. The total follow-up duration of this trial will be two years. Comparison of changes in primary outcomes, comparison of change in secondary outcomes, as well as comparison of safety outcomes, will be done between the treatment group and control group.

Outcomes

Primary outcomes include changes in axial length (AL) and changes in cycloplegic SER. The SER is calculated from the dioptric powers of the sphere and half of the cylinder (sphere + 0.5 *cylinder).

Secondary outcomes include changes in the following indicators, including choroidal thickness (ChT), intra-ocular pressure, flat keratometry (K1), and steep keratometry (K2), central corneal thickness, anterior chamber depth, and length thickness.

Safety outcomes refer to the fundus related examinations including fundus photography, and optical coherence tomography (OCT). The changes in uncorrected distance visual acuity (UDVA) will also be treated as a safety outcome, reflecting the functional status of the retina.

All the above outcomes will be measured at the baseline and every six months. For children in the treatment group, there will an additional measure, a telephone questionnaire that asks about the afterimage time after device use. Subjects will be telephoned twice between each follow-up time point, at an interval of more than one month.

Outcomes Measurements

Refractive errors will be measured using an autorefractor (ARK-510A; Nidek Co. Ltd, Aichi, Japan). All children will be pupil dilated with cycloplegic before autorefraction measurement.

Measurements of AL, central corneal thickness, anterior chamber depth, length thickness, K1, and K2 will be done with an optical biometer (Lenstar LS 900; HAAG-STREIT AG, Switzerland).

Measurements of intraocular pressure were done with a non-contact tonometer (Canon TX-20; Canon Inc., Tokyo, Japan). ChT will be measured with an optical coherence tomography (Spectralis HRA+OCT, Heidelberg Engineering, Germany) at nine locations of the fundus as follows: subfoveal, 1mm and 3mm in the temporal side of the fovea, 1mm and 3mm in the nasal side of the fovea, 1mm and 3mm in the superior side of the fovea, 1mm and 3mm in the inferior side of the fovea. Both baseline measurement and follow-up measurements will be done by the same group of trained investigators.

Benefits of this study

For children participating this trial, they will receive professional guidance on myopia intervention by ophthalmologists from Beijing Tongren Hospital. For children in the treatment group, they could use our intervention equipment for free. This study may bring more benefits in the future, as myopia is a global public health burden. The 650 nm low-level red-light intervention may help decrease the myopia incidence, slow down the myopia progression.

References:

[1]. Dolgin E. The myopia boom. Nature. 2015;519(7543):276-278.

[2]. Wei S, Sun Y, Li S, Hu J, Yang X, Lin C, et al. Refractive Errors in University Students in Central China: The Anyang University Students Eye Study. Invest Ophthalmol Vis Sci. 2018;59(11):4691-4700.

[3]. Foo LL, Lanca C, Wong CW, Ting D, Lamoureux E, Saw SM, et al. Cost of Myopia Correction: A Systematic Review. Front Med (Lausanne). 2021;8:718724.

[4]. Fernandez-Vega-Cueto L, Alfonso-Bartolozzi B, Lisa C, Madrid-Costa D, Alfonso JF. Seven-year follow-up of posterior chamber phakic intraocular lens with central port design. Eye Vis (Lond). 2021;8(1):23.

[5]. Vrijman V, van der Linden JW, van der Meulen I, Mourits MP, Lapid-Gortzak R. Multifocal intraocular lens implantation after previous corneal refractive laser surgery for myopia. J Cataract Refract Surg. 2017;43(7):909-914.

[6]. Vargas V, Alio JL. Refractive outcomes and complications following angle supported, iris fixated, and posterior chamber phakic intraocular lenses bilensectomy. Curr Opin Ophthalmol. 2021;32(1):25-30.

[7]. Krueger RR, Meister CS. A review of small incision lenticule extraction complications. Curr Opin Ophthalmol. 2018;29(4):292-298.

[8]. Leccisotti A, Fields SV. Diffuse lamellar keratitis after LASIK with low-energy femtosecond laser. J Cataract Refract Surg. 2021;47(2):233-237.

[9]. Wang Y, Ma J, Zhang L, Zou H, Li J, Zhang Y, et al. Postoperative Corneal Complications in Small Incision Lenticule Extraction: Long-Term Study. J Refract Surg. 2019;35(3):146-152.

[10]. Blum M, Lauer AS, Kunert KS, Sekundo W. 10-Year Results of Small Incision Lenticule Extraction. J Refract Surg. 2019;35(10):618-623.

[11]. Belin MW, Khachikian SS. An introduction to understanding elevation-based topography: how elevation data are displayed - a review. Clin Exp Ophthalmol. 2009;37(1):14-29.

[12]. Chua D, Htoon HM, Lim L, Chan CM, Mehta JS, Tan D, et al. Eighteen-year prospective audit of LASIK outcomes for myopia in 53 731 eyes. Br J Ophthalmol. 2019;103(9):1228-1234.

[13]. Kamiya K, Igarashi A, Hayashi K, Negishi K, Sato M, Bissen-Miyajima H. A Multicenter Prospective Cohort Study on Refractive Surgery in 15 011 Eyes. Am J Ophthalmol. 2017;175:159-168.

[14]. Tran H, Tran YH, Tran TD, Jong M, Coroneo M, Sankaridurg P. A Review of Myopia Control with Atropine. J Ocul Pharmacol Ther. 2018;34(5):374-379.

[15]. Jiang Y, Zhu Z, Tan X, Kong X, Zhong H, Zhang J, et al. Effect of Repeated Low-Level Red-Light Therapy for Myopia Control in Children: A Multicenter Randomized Controlled Trial. Ophthalmology. 2021.

6. Data management and statistical analysis

Data collection and data cleaning

All measurement, no matter the baseline or the follow-up measurements, will be done by the same group of trained investigators under the same guidelines to ensure the data quality. All the examination data will be collected using self-designed clinical research data collection form, and will be independently entered into the database using Epidata software 3.1 (The Epidata Association, Odense, Denmark) by two research associates.

There will be a data cleaning process before data analysis, this will be done for the logical error checking, and the missing data imputation. The open-source R program (https://www.r-project.org/, version 4.0.4) will be used for data cleaning.

Missing data handling

Some subjects may loss to follow-up during the trial. For the primary outcomes, secondary outcomes, and the safety outcomes, the missing data at each follow-up time point will be imputed using a Markov Chain Monte Carlo（MCMC）method.

Statistical analysis

Data-set that used for analysis

Data analysis will be performed for all randomly assigned children by means of an intention-to-treat (ITT) method, before which the MCMC imputation will be done. The conclusions of this study will be drawn based on the results from ITT analysis.

At the same time, this trial will also provide the results analyzed based on the Per-protocol (PP) data-set. The PP data-set will exclude children that are lost to follow-up or who discontinue the intervention.

Statistical description and hypothesis testing

The mean values and standard deviations will be used for the statistical description of continuous variables (e.g. age), while frequency and percentage will be used for the statistical description of the categorical variables (e.g. gender). The T-test will be used for the comparison of continuous variables between two groups, while the Chi-square test or Fisher exact test will be used for comparison on categorical variables between two groups. Pearson correlation analysis will be used to explore the association between change in AL, change in SER, and change in ChT. The strength of correlation can be divided into three levels according to the correlation coefficient (r): Strong, $r \geq 0.7$. Moderate, $0.7 > r \geq 0.4$. Weak, $r < 0.4$. In addition, data analyses will be based on data from the children's right eyes, except for the definition of low, moderate and high myopia, which will be defined by person.

Interim analysis

There will be two interim analysis, at 6-month follow-up, and 12-month follow-up, the significance level would be adjusted to be 0.0225 after O'Briene Fleming α-spending adjustment.

Subgroup analyses

In order to evaluate whether the 650 nm low-level red-light has the same intervention effect on myopic and non-myopic children, each group of children will be further divided into two subgroups according to whether they are myopic at enrollment (subgroup TM: children that are myopic at baseline in the

treatment group; subgroup TN: children that are non-myopic at baseline in the treatment group; subgroup CM: children that are myopic at baseline in the control group; subgroup CN: children that are non-myopic at baseline in the control group.)

And in some condition, participants will be stratified by age and gender to assess whether gender or age has an interaction effect on the treatment effect.

Statistical Software

All analysis will be completed with the open-source R program (https://www.r-project.org/, version 4.0.4). The significance level is 0.05, two-tailed.

7. Ethical consideration

Institutional Review Board Review and Informed Consent

This protocol, together with the informed consent form, and enrollment files (e.g. the advertisement on Wechat APP) will be reviewed by the Institutional Review Board (IRB) of Beijing Tongren Hospital, Capital Medical University. Written informed consent would be obtained from children's parents, this clinical trial will adhere to the tenets of the Declaration of Helsinki. This study will not starts unless the ethic approval was obtained.

Subject Confidentiality

All examinations, evaluation forms, reports, and other records that leave the site will be identified by coded number only to maintain subject confidentiality. All records will be kept locked. All computer entry and networking programs will be done with coded numbers only. Clinical information will not be released without written permission of the children's parents (or legal guardian), except as necessary for monitoring by the IRB.
