# Supplementary material for: Daily Low-Level Red Light for Spherical Equivalent Error and Axial Length in Children With Myopia: A Randomized Clinical Trial
Source: JAMA Ophthalmol. 2024 Apr 25;142(6):560–7. doi: 10.1001/jamaophthalmol.2024.0801 (PMC11046409; doi:10.1001/jamaophthalmol.2024.0801)
Supplement: Supplement 3. — Data Sharing Statement [file jamaophthalmol-e240801-s003.pdf]

Data Sharing Statement

Cao. Daily Low-Level Red Light for Spherical Equivalent Error and Axial Length in Children With Myopia. *JAMA Ophthalmol*. Published April 18, 2024.
doi:10.1001/jamaophthalmol.2024.0801

Data
Data available: No
